# Supramolecular Organization As a Factor of Ribonuclease Cytotoxicity

**DOI:** 10.32607/actanaturae.11000

**Published:** 2020

**Authors:** E. V. Dudkina, V. V. Ulyanova, O. N. Ilinskaya

**Affiliations:** Institute of Fundamental Medicine and Biology, Kazan (Volga Region) Federal University, Kazan, 420008 Russia

**Keywords:** ribonuclease, dimer, oligomerization, catalytic activity, cytotoxicity, antitumor activity

## Abstract

One of the approaches used to eliminate tumor cells is directed destruction/modification of their RNA molecules. In this regard, ribonucleases (RNases) possess a therapeutic potential that remains largely unexplored. It is believed that the biological effects of secreted RNases, namely their antitumor and antiviral properties, derive from their catalytic activity. However, a number of recent studies have challenged the notion that the activity of RNases in the manifestation of selective cytotoxicity towards cancer cells is exclusively an enzymatic one. In this review, we have analyzed available data on the cytotoxic effects of secreted RNases, which are not associated with their catalytic activity, and we have provided evidence that the most important factor in the selective apoptosis-inducing action of RNases is the structural organization of these enzymes, which determines how they interact with cell components. The new idea on the preponderant role of non-catalytic interactions between RNases and cancer cells in the manifestation of selective cytotoxicity will contribute to the development of antitumor RNase-based drugs.

## INTRODUCTION

Ribonucleases (RNases) catalyze the cleavage of phosphodiester bonds in various RNA substrates, playing a key role in the degradation and processing of cellular RNA [1]. Most of the known RNases are proteins; however, atypical RNase forms have also been encountered, the catalytic part of which is represented by an RNA molecule. Therefore, RNases are some of the few enzymes that have apparently retained a connection with the initial world of RNAs, an ancient system of RNA replicators and catalysts [1].

RNases are classified into exo- and endoribonucleases. Exoribonucleases catalyze the 3’ → 5’ hydrolysis of the phosphodiester bond situated between nucleotides located at the polynucleotide chain ends. Endoribonucleases cleave phosphodiester bonds within single-stranded or double-stranded RNAs.

The cells of living organisms contain various types of exo- and endoribonucleases, the main function of which is to control gene expression via changing the stability of various RNA types and eliminating unnecessary intracellular RNAs [2]. In addition, by cleaving foreign RNAs that have penetrated the cell [3] and participating in cellular suicide, RNases play a protective role [4].

Secreted RNases of microorganisms perform digestive, protective, and regulatory functions. They are required for RNA hydrolysis in the extracellular space. The cleavage of extracellular RNA in microorganisms is believed to occur mainly for extracting nutrients. Only a few reports have indicated involvement of the secreted RNases of microorganisms in the competition for an ecological niche [5], implementation of the pathogenic potential [6–8], and defense of their population and associated organisms from viral infection [9, 10].

In higher organisms, secreted RNases, on the contrary, are less involved in food digestion and are components of the innate system for defense and physiological homeostasis maintenance. In plants, they determine self-incompatibility [11]. In vertebrates, secreted RNases hydrolyze the extracellular RNA released from damaged, stress-induced, or malignant cells, thereby exerting anti-inflammatory and anticoagulant effects, and possessing antimicrobial and antiviral activities, as well as immunomodulatory and regenerative properties [12].

Certain types of secreted RNases in animals are involved in tumorigenesis [13], while others suppress the proliferation of cancer cells and induce apoptosis in them [14-19], which makes RNases potential antitumor agents in the sparing therapy of malignant neoplasms. Selective cytotoxicity towards tumor cells is also exhibited by the microbial RNases [18-22] that are insensitive to the mammalian RNase inhibitor (RI), which opens up wide perspectives for bioengineering [23]. RNases can be internalized by cells via receptor-dependent endocytosis in order to regulate signaling pathways and intracellular RNAs [13]. In this case, the ribonucleolytic activity is not always of primary significance; probably, the key role is played by the physicochemical and structural properties of these proteins.

## SECRETED RIBONUCLEASES OF BACILLI

Among the extracellular bacterial RNases exhibiting antitumor activity, secreted RNases of bacilli have been described in detail [19, 20, 22, 24, 25]. Bacillary RNases are represented by two types of endonucleolytic enzymes: low-molecular-weight guanyl-preferring RNases [24] and high-molecular-weight nonspecific RNases [26, 27]. High-molecular-weight bacillary RNases (binase II, RNase Bsn), members of the HNH endonuclease family (IPR003615), consist of about 240 amino acid residues (30 kDa). These proteins are stable in a pH range of 6.5–9.5, have an isoelectric point of about 5, and non-specifically cleave RNA to form 5’-phosphorylated oligonucleotides. For catalytic activity, they require Mg^2+^ ions. For RNA hydrolysis, the optimum pH is 8.5 and the optimum temperature is 37°C.

Low-molecular-weight guanyl-preferring bacillary RNases (binase, barnase), who are members of the N1/T1/U2 family (IPR000026), are small extracellular proteins consisting of approximately 110 amino acid residues (12 kDa). The enzymes are stable over a wide pH range (3–10). Guanyl-specific RNases are cationic proteins with an isoelectric point of about 9. They catalyze the cleavage of RNA, preferably at guanosine residues, in two successive reactions during which transesterification of the 5’-phosphoether bond leads to the formation of cyclic 2’, 3’-phosphodiesters as intermediate hydrolysis products, which are subsequently cleaved to nucleoside 3’-phosphates [28]. For catalytic activity, these enzymes do not require metal ions or cofactors [29]. The optimal conditions for RNA hydrolysis are pH 8.5 and a temperature of 37°C.

The synthesis of extracellular RNases in bacilli is induced, with rare exceptions, under phosphate starvation conditions [30, 31], while that of low-molecular-weight RNases is also induced under nitrogen starvation conditions [32], which indicates how significant these enzymes are in providing cells with nutrients. It should be noted that the RNase activity level of low-molecular-weight RNases is 1–2 orders of magnitude higher than that of high-molecular-weight RNases. Low-molecular-weight RNases also have the specific features of the ribonucleolytic reaction mechanism: preference for guanyl residues, formation of the cyclic 2’, 3’-ribonucleotides present in the reaction medium for at least 1 h [33], and a phosphate group at the 3’ end of the formed nucleotides. Currently, 2’, 3’-cycloderivatives of the nucleotides found in both pro- and eukaryotes are considered in eukaryotes as components of the pathway that protects tissues from infection and damage [34]. Nucleotides with a 5’-terminal phosphate can be ligated to similar nucleotides to form polymeric structures, while insertion of a nucleotide with a 3’-terminal phosphate requires additional reactions to transfer the phosphate group to the 5’-end. These features, along with the fact that high-molecular-weight RNases abund in the bacterial world, and that low-molecular-weight RNases are present only in a limited number of bacterial species [35], make low-molecular-weight RNases of bacilli unique proteins and suggest that they have special functions and biological properties.

For example, there is evidence that indirectly indicates the antagonistic properties of low-molecular-weight RNases [5, 24] and their involvement in the protection of bacterial cells from phage infection [9]. In pathogenic bacilli from the *Bacillus cereus *group, low-molecular-weight RNases are involved in surface toxins [35]. To date, various biological effects, from growth-stimulating to antiproliferative, of the low-molecular-weight RNases of bacilli have been demonstrated [19, 20, 22, 36, 37], which makes them promising for practical use. The potential of the high-molecular-weight RNases of bacilli has not yet been explored.

The low-molecular-weight RNases of bacilli have a high degree of primary structure similarity (more than 73%); the main differences occur in the regulatory regions of the genes, which results in different production levels of these proteins, as well as in signal peptides that affect their secretion [35]. The enzymes have an almost identical tertiary structure and possess general physicochemical and catalytic properties. The amino acid residues His and Glu in the enzyme active site act as common acid-base groups during catalysis, and the Arg and Lys residues are important for phosphate binding.

The first studies on the isolation and purification of low-molecular-weight RNases were conducted in the 70s: *B. amyloliquefaciens *RNase (barnase) and *B. pumilus *RNase 7P (binase) were isolated and characterized [38, 39]. We have improved a method for the isolation of bacillary RNases which enables preparation of a homogeneous protein in three stages. This method was used to isolate, chromatographically purify, and characterize guanyl-preferring RNases from *B. pumilus *7P (binase), *B. altitudinis *B-388 (balnase), and *B. licheniformis *(balifase) [30, 40, 41]. Among the presented species, the most active RNase producer is *B. pumilus *secreting binase. For a long time, *B. amyloliquefaciens *ribonuclease (barnase) was believed to be a close homologue of binase. The similarity of the primary structures of binase and barnase is 85%; however, the synthesis of barnase is not subject to phosphate regulation but depends on the multifunctional protein Spo0A [24].

Investigation of a new RNase, balnase, secreted by the *B. altitudinis *B-388 strain has demonstrated that it is the closest natural homologue of binase. The primary structures of the proteins differ only in one amino acid substitution: threonine at position 106 in the binase molecule is replaced by alanine in balnase [29], which does not affect the isoelectric point of the protein but somewhat reduces its thermal stability [29, 42].

*The B. licheniformis *RNase balifase has a primary structure similar to that of binase (73%) and barnase (74%). Balifase synthesis is induced under phosphate starvation conditions, which brings the enzyme closer to binase and balnase, but the physico-chemical properties of balifase are closer to those of barnase [41].

Despite the fact that secreted RNases of bacilli are similar in their physico-chemical and catalytic properties, they differ in their dimerization mode and stability of dimeric forms, which affects the cytotoxic properties of these RNases.

**RNase oligomerization**

Oligomerization is one of the most common phenomena, and a key factor, in the regulation of enzymes, ion channels, receptors, and transcription factors. Dimers and oligomers ensure the stability of proteins, activate signal transduction across the membrane, enhance enzymatic activity, and expand the possibilities for regulation, providing combinatorial specificity, allosteric properties, activation, and inhibition of the catalytic activity of enzymes [43].

Investigation of the structural organization of the RNases isolated by us – binase, balnase, and balifase – has revealed that all of them dimerize *in vivo *and are natural dimers [41, 44, 45]. Probably, the formation of RNase dimers is one of the key processes necessary for the enzymes to perform their functions and manifest their biological properties. Despite their high degree of structural similarity, the dimerization mode and stability of dimeric structures in homologous RNases are very different [22].

We have identified, for the first time, the natural dimeric structures of binase that had been known for a long time as a monomer incapable of oligomerization [44]. Previously, binase dimers had been found only in a protein crystal [46]. The theoretical possibility of enzyme dimerization in solution was considered an artifact that can occur only at a high protein concentration [47]. We have shown that binase *in vivo *occurs in two dimeric forms differing in their mechanism of formation and stability. Some binase dimers are highly stable, apparently due to the exchange of N- or C-terminal regions, and do not dissociate under denaturing conditions; others are incapable of exchanging domains between monomers (swapping interactions), which leads to the dissociation of these dimers into monomers during electrophoresis under denaturing conditions [44]. Balnase and balifase constitute only the second type of dimers [22, 41, 45].

Molecular modeling of the dimeric structures of binase, balnase, and balifase revealed a variety of dimers (*Figure*). It should be noted that bacillary RNase dimers are stabilized by non-covalent bonds, because the primary protein structures lack sulfur-containing amino acids [48]. Given the forces involved in the protein complex formation (electrostatic, hydrophobic, van der Waals, electrostatic, or their balance), two models in each group were selected (*Figure*). It is noted that binase is able to form four dimer types (*Fig A*), while balnase (*Fig B*) and balifase (*Fig C*) form three and two types, respectively, with one of the types being a variant with a blocked enzyme active site.

**Figure:**
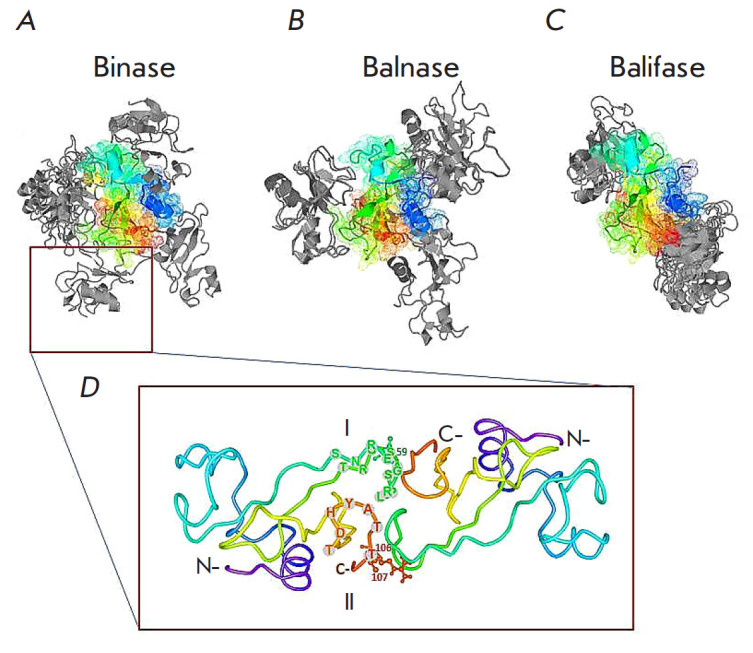
Models of bacillary RNase dimers. Modeling of the protein-protein interaction of RNase monomers was performed by the direct method through a search for structures with minimum Gibbs free energy. The models are classified into groups, based on the forces involved in the protein complex formation (electrostatic, van der Waals and electrostatic, hydrophobic, or their balance); two structures with the lowest free energy are selected from each group. One of the monomers of binase (*A*), balnase (*B*), or balifase (*C*) is presented as a molecule with secondary structure elements shown in rainbow colors, from the N-terminus (blue) to the C-terminus (red). The potential positions of the second monomer in RNase dimers are shown in gray. (*D*) The unique binase dimer that is absent in balnase and balifase. The contact surface in the dimer is formed by two flexible loops I (amino acid residues 56–69) and II (amino acid residues 99–104) [57] which enable the monomers to exchange C-terminal regions

An analysis of the mechanisms of bacillary RNase dimerization raises the question of active site accessibility for substrate hydrolysis in dimer molecules. The investigation of a binase crystal revealed that the RNA in the dimer is bound to only one of the two monomer molecules, because the catalytic site of the second subunit is blocked in the dimeric structure [21]. Mutant binase Glu43Ala/Phe81Ala has a higher catalytic activity and more pronounced cytotoxic properties towards Kasumi-1 leukemia cells compared to those of the wild-type enzyme, which is associated with the inability of the mutant to form self-inhibiting dimeric structures [49].

A Brownian dynamics simulation demonstrated that binase forms three dimer types, depending on the active site accessibility [50]. Dimeric structures of the first-type have two open catalytic sites that are involved in RNA hydrolysis. In dimers of the second and third types, one or both active sites are blocked. An analysis of the monomer association rate during binase dimerization showed that the rate constant of the first type dimer formation is much higher than that in models of the second and third types, and its value is comparable to the rate of binase and barstar inhibitor complex formation [50]. Given the similar levels of catalytic activity of binase, balnase, and balifase, as well as the results of the analysis of the protein emission band intensity and the area of hydrolysis zones, we can state that both active sites in the dimer molecules of the studied RNases are involved in catalysis [22] and that dimers with partial or completely closed active sites appear to be minor.

It should be noted that most of the dimers found in nature form through non-covalent bonds between extracellular domains, transmembrane regions, and/or N, the C-termini of proteins [51]. The last mechanism can occur in two ways. The first is contact dimerization, when the loop of one of the monomers forms stabilizing contacts with another molecule; the second is terminal domain exchange or domain swapping [51]. Domain exchange is typical of proteins such as cytochrome *c *[52] and, in particular, some amyloidogenic proteins, such as human prion protein, cystatin C, or β_2_-microglobulin [53, 54].

The phenomenon of domain exchange partially contradicts Anfinsen’s dogma that the amino acid sequence determines the unique protein tertiary structure [55]. In fact, flexible loops of the protein can occur in variable conformations, occupying more than one available energy minimum [56]. This enables domains connected to flexible protein regions to occur in different orientations and to interchange with an equivalent domain of the neighbor subunit. Therefore, the presence of more than one flexible loop enables the formation of non-covalent dimers or larger oligomers, which gives enzymes new opportunities for allosteric interactions and macromolecular signaling [57, 58]. In binase, two flexible loops are located around the active site: the first loop is formed by the amino acid residues 56–69, and the second is formed by the amino acid residues 99–104 [59]. Both loops occur in close proximity in a binase dimer variant that is absent in other RNases (*Fig D*). It is stabilized by Phe105, Thr106, Arg107, Glu59, and Gly60. Thr106 is the only amino acid residue changed in the balnase molecule in comparison with binase. Replacement of polar threonine with hydrophobic alanine affects the stability of balnase [22, 29, 42]. There may be an exchange of C-terminal regions during the formation of a stable binase dimer. The lack of such a mechanism in balnase and balifase leads not only to significant differences in the ways of their dimerization compared to binase, but also to a decrease in the stability of the dimers and the antitumor potential of homologous RNases [22].

To date, several RNases have been identified. Their functionality depends on the structural organization of their molecules. For example, the antiviral potential of RNase L and monocyte chemoattractant protein-1-induced protein 1 (MCPIP1) is initiated by the formation of dimeric structures [60, 61]. Among animal RNases, bovine seminal RNase (BS-RNase), which is a natural dimer, is the most fully characterized [62]. There is a correlation between the efficiency of catalysis and dimerization of microbial RNase T from *Escherichia coli *[63]. *B. subtilis *RNase J functions in a cell as a dimer or higher-order oligomer [64].

For a long time, among the diversity of RNases, only one natural dimer capable of domain exchange had been known—BS-RNase, a mixture of two dimer types [65]. Some dimeric structures form through the covalent disulfide bridges that exist between the amino acid residues Cys31 and Cys32; dimers of the second type are additionally stabilized thanks to the interchange of the N-terminal α-helices of the enzyme [66]. Only second-type dimers appear to exhibit antitumor activity. The possibility of domain exchange leads to the formation of highly stable dimeric structures that are not destroyed during the penetration of the enzyme into the cell and remain insensitive to the action of RI, exhibiting their cytotoxicity via the hydrolysis of intracellular RNA [65].

Another RNase whose dimer is capable of domain swapping is pancreatic RNase A [67]. The enzyme is able to self-associate non-covalently upon interaction with a substrate as well as oligomerize upon lyophilization in 40% acetic acid [68, 69]. Dimers and higher-order oligomers form through an exchange of the domains involving the N- and/or C-termini of the protein [70]. Swapping oligomers of RNase A increase their enzymatic activity towards double-stranded RNA (dsRNA) or DNA:RNA hybrids compared to that of the native monomer [71]. The increase in the catalytic activity is directly proportional to the size of the oligomer; furthermore, species containing more C-swapping oligomeric structures than N-swapping ones exhibit the highest enzymatic activity because of the higher basicity of the C-oligomer charge [72]. Contradictory results were obtained in a study of the antitumor potential of RNase A oligomers, which requires further research.

Onconase, RNase of the leopard frog *Rana pipiens*, is also capable of swapping dimerization. The enzyme forms dimeric structures through the exchange of N-terminal fragments during lyophilization in 40% acetic acid [73]. In this case, the C-terminus of the enzyme is unable to proceed with the exchange because it is blocked by the disulfide bond between Cys87 and Cys104 [58]. Dimerization of onconase enhances its biological activity, as in other RNases [17, 74, 75]. For example, the onconase dimer was found to be more cytotoxic for pancreatic cancer cells than the native monomer [73]. Enhancing of cytotoxicity during dimerization is associated with an increase in the basicity of the onconase molecule, which enhances the enzyme’s affinity to the negatively charged membranes of cancer cells and/or their intracellular targets [75, 76].

RNase oligomerization protects from RI and increases the molecular charge, improving the internalization of the enzyme into tumor cells; it increases the enzymatic activity of RNases and their affinity to dsRNA [62, 70]; and it provides RNases with new biological properties [65, 70] or enhances existing ones. Therefore, the ability of RNases to form oligomeric structures by means of the domain-swapping mechanism is central to their cytotoxicity.

**Antitumor RNase activity**

RNases exhibit selective cytotoxicity towards certain cancer cells without significantly affecting the normal cells of the body, which makes these enzymes a potential alternative to modern anticancer drugs [20, 24, 25].

The most prominent bacterial RNase, binase, exerts an antiviral effect on influenza A (H1N1), rabies, the foot and mouth disease, and several plant viruses [77]. Binase exhibits selective cytotoxicity towards tumor cells expressing certain oncogenes: *ras*, *KIT*, *AML/ETO*, *FLT3*, *E6*, and *E7 *[18, 19, 21]. Despite the active investigation of RNase selectivity, the mechanism of RNase selective action still remains unclear.

The biological effects of RNases are mediated by the molecular determinants that contribute to the apoptosis-inducing effect of enzymes, which include catalytic activity, the structure and charge of the molecule, and its stability [25]. However, little attention has been paid to the contribution of supramolecular organization to RNase cytotoxicity.

For a long time, the decisive role in RNase cytotoxicity was believed to be played by their enzymatic activity [78]. However, there is increasing evidence that enzymes lacking catalytic activity are also able to induce the death of tumor cells. Mutant forms of α-sarcin and the human eosinophil cationic protein, which are incapable of RNA hydrolysis, have been shown to retain their toxicity and trigger apoptosis in cancer cells [79, 80]. The antitumor activity of the human eosinophil cationic protein is due to its interaction with the surface structures of the cell, which changes the permeability of the plasma membrane and disrupts the ionic equilibrium without internalization of the enzyme or hydrolysis of intracellular RNA [81]. RNase A and its homologues were found to be capable of binding to dsRNAs without exhibiting catalytic activity, probably affecting the regulatory functions of these molecules [20]. The high affinity of RNase A for dsRNA is due to the positively charged amino acids located near the active site [82]. Bacterial RNase III contains two separate domains, one of which binds to dsRNA, and the other destructs dsRNA [83]. According to the data presented, the enzyme regulates gene expression either by cleaving dsRNA or by binding to it, which leads to functional changes in the dsRNA molecule [83].

Although treatment of cells with binase leads to a decrease in the intracellular RNA level, this process is not directly associated with the induction of apoptosis [84]. A decrease in the amount of total RNA is accompanied by an increase in the expression of the pro-apoptotic genes *p53 *and *hSK4 *1.5- and 4.3-fold, respectively, while the mRNA level of the anti-apoptotic gene *bcl-2 *decreases 2-fold. Probably, hydrolysis of RNA substrates by binase triggers a cascade of reactions that regulate the genes that control apoptosis [84]. Also, there is no direct correlation between a decrease in the RNA level and the toxic effect of RNases. For example, in Kasumi-1 acute myeloid leukemia cells, which are extremely sensitive to binase, the total RNA level did not change even when the viability was decreased by 95% [85]. Onconase induces the apoptosis of mitogen-stimulated lymphocytes without affecting the level of intracellular RNA [86].

Today, the primary interaction between RNases and surface cell structures is considered one of the most significant processes that play an important role in the triggering of a cascade of reactions leading to the death of tumor cells. Internalization of RNases occurs either through specific interaction with cell receptors [87] or through their direct interaction with the cell membrane [76]. RNases interact with the target cell surface through the involvement of membrane lipids, ion channels, and receptors, as well as through nonspecific electrostatic binding [88]. Native and mutant dimeric RNases were shown to strongly affect aggregation, fluidity, and the fusion of cell membranes [75]. RNase A and its analogue, human pancreatic ribonuclease (RNase 1), were found to specifically interact with neutral hexasaccharide glycosphingolipid Globo H [88] located on the outer side of the epithelial cell membrane and present in large amounts in some tumor cells [89]. Onconase and BS-RNase interact with specific non-protein receptor-like molecules on the plasma membrane, which is not typical of other RNases [90].

One of the mechanisms underlying the selective cytotoxicity of binase and other cationic RNases is the ability of RNases to interact with the anionic groups on the surface of cancer cells [25]. Tumor cells are known to be more electronegative than normal cells due to a high content of acidic phospholipids [91]. Enzyme dimerization leads to an increase in the cationicity of the protein and, therefore, to the enhancement of their antitumor properties. For example, replacement of negatively charged amino acid residues on the surface of *Streptomyces aureofaciens *RNase (RNase Sa) with positively charged ones increased the cytotoxic potential of the enzyme [92, 93]. The apoptosis-inducing effect of RNase Sa on Kasumi-1 acute myeloid leukemia cells significantly correlated with an increase in the enzyme cationicity [18]. Introduction of positively charged residues into the amino acid sequence of the protein increased onconase cytotoxicity [94].

However, increasing the charge alone was found not to be enough for a successful internalization of RNases into the cell. The extremely important role of the specific orientation of the RNase molecule (onconase, BS-RNase, RNase 1, and RNase A) relative to the cell membrane was demonstrated [76]. For example, native dimeric BS-RNase adopts the most favorable orientation for its internalization when it points both of its N-termini towards the cell membrane [75]. The Gly38Lys BS-RNase mutant with an additional cationic residue oriented towards the N-terminus interacted with the membrane more strongly and was more cytotoxic than wild-type BS-RNase [17]. The presented data once again demonstrate the importance of the three-dimensional structure of RNases, in particular the orientation of the main charges that affect the cytotoxic potential of these enzymes.

Binase causes the death of the murine-transformed lung epithelial cells MLE-12, without significantly affecting normal AT-II cells [95]. In this case, after 24-h incubation, binase reaches the nucleus of AT-II cells without exerting any cytotoxicity and causes the death of MLE-12 cells without penetrating them [95]. How does RNase mediate its cytotoxic potential without internalization of the enzyme? This question remained unanswered for a long time.

We recently found that the selectivity of binase for tumor cells expressing the *ras *oncogene was due to the direct interaction of RNase with the endogenous protein KRAS [96]. Investigation of activated KRAS using a non-hydrolyzable analogue of GTP (GTPγS) showed that binase prevents the exchange of GDP for GTP and reduces the interaction between RAS and the protein factors GEF and SOS1. An analysis of the phosphorylation of RAS effectors, the AKT and ERK1/2 proteins, confirmed the inhibition of the MAPK/ERK signaling pathway [96]. Therefore, the selectivity of binase for tumor cells expressing the *ras *oncogene was proven to be associated with the interaction between binase and KRAS, which leads to blockage of the MAPK/ERK signaling pathway and triggering of apoptosis in tumor cells. KRAS-bound binase is found not only in dimeric form, but also in trimeric form, which confirms the importance of enzyme aggregation into higher-order oligomers for blocking proliferative signals [96].

RNase A is also capable of affecting cellular signals, but its action is opposed to the antitumor effect of binase. The enzyme interacts with the epidermal growth factor receptor (EGFR) and activates the MAPK/ERK signaling pathway, which leads to the induction of cell proliferation and tumor growth [13]. This feature of RNase A, which was discovered relatively recently, compromises the possibility of using this enzyme as a potential antitumor agent.

Some RNases have to enter the cell to exert their cytotoxic potential. Conflicting data on the mechanism of RNase internalization have been reported. For example, onconase and RNase A are internalized in early endosomes of HeLa and K562 cells via clathrin- and caveolin-independent pathways [87], while endocytosis of onconase in Jurkat cells occurs in a dynamin-dependent way [97]. These conflicting data suggest that RNases can use different pathways to enter cells, while many aspects of RNase internalization still remain unknown. BS-RNase is internalized in the endosomes of both normal and malignant cells, but only in the latter, where the enzyme is cytotoxic, does it reach the Golgi complex that ensures its cytosolic delivery [90]. A BS-RNase variant the C-terminus of which is designed for localization in the endoplasmic reticulum lacks cytotoxicity because it cannot be released in the cytosol to exert its antitumor activity [90].

Upon reaching the cytosolic compartment, RNases encounter another obstacle; the intracellular mammalian ribonuclease inhibitor. RI is a 50-kDa protein that is present in the cytoplasm, mitochondria, and the nucleus of animal and human cells [98]. The biological functions of RI have not yet been fully elucidated; RI is considered to be potentially involved in cell redox homeostasis [99]. RI blocks mammalian RNases by forming tight complexes with them, which inhibit their catalytic activity. The phylogenetic remoteness of bacterial RNases and amphibian RNases underlies their insensitivity to RI and makes them potential antitumor agents. BS-RNase is insensitive to RI due to natural dimerization, forming three-dimensional structures that are inaccessible for blockage by the inhibitor. Also, as mentioned earlier, only dimers stabilized by domain exchange are insensitive to RI and exhibit cytotoxicity [65], which once again emphasizes the significance of RNase oligomerization.

The use of homologous RNases to study the dimer formation mechanism allowed us to discover the contribution of dimeric structure stability to the manifestation of the antitumor potential of these enzymes. Investigation of the cytotoxic effect of balnase and balidase on the human lung adenocarcinoma cells A549 has demonstrated that binase has the most pronounced apoptogenic effect, and that its cytotoxic potential enhances as the duration of incubation with cells increases, while the activity of balnase and balifase begins to decrease after 48 h of incubation [22]. These data are an indication of the key role of the stability of dimeric structures in enzyme cytotoxicity. Balnase and balifase dimers, in contrast to binase dimers, are less stable due to their inability to domain-exchange; after 48 h, they probably dissociate into monomers, which decreases their toxic properties. Dimeric binase structures are highly stable and can induce the death of tumor cells for a long time [22].

The presented information indicates that the antitumor activity of RNases is the result of a complex interaction between the structural and functional features of the enzymes, and that RNase oligomers have a higher cytotoxic potential than monomers [62, 70].

The cytotoxic effect of RNases is known to be associated not only with the consequences of direct RNA degradation, but also with the regulatory effects of its hydrolysis products [20, 86]. The manifestation of the biological effects of RNases is related to various cellular mechanisms, including the non-catalytic interaction between RNases and cellular components, the internalization of the proteins into the cell, and the ability to avoid RI action. Each cytotoxic RNase type has its own specific set of molecular mechanisms which mediates the antitumor effect of the enzyme, but the defining one among them is the structural organization of RNase molecules, which contributes to each of the presented molecular mechanisms.

The results of our study have revealed a direct correlation between cytotoxicity and the stability of dimeric RNase structures, confirming the fundamental role played by the supramolecular organization of enzymes in their antitumor activity.

## Abbreviations

dsRNA: double-stranded RNA
RI: mammalian ribonuclease inhibitor
BS-RNase: bovine seminal ribonuclease
